# On the Compatibility of Fish Meal Replacements in Aquafeeds for Rainbow Trout. A Combined Metabolomic, Proteomic and Histological Study

**DOI:** 10.3389/fphys.2022.920289

**Published:** 2022-06-29

**Authors:** Antonio Palomba, Riccardo Melis, Grazia Biosa, Angela Braca, Salvatore Pisanu, Stefania Ghisaura, Christian Caimi, Ilaria Biasato, Sara Bellezza Oddon, Laura Gasco, Genciana Terova, Federico Moroni, Micaela Antonini, Daniela Pagnozzi, Roberto Anedda

**Affiliations:** ^1^ Porto Conte Ricerche S.r.l, Alghero (SS), Italy; ^2^ Department of Agricultural, Forest and Food Sciences, University of Turin, Grugliasco (TO), Italy; ^3^ Department of Biotechnology and Life Sciences, University of Insubria, Varese, Italy

**Keywords:** hepatic metabolism, insect meal, NMR-based metabolomics, poultry by-products meal, proteomics, sustainable aquaculture

## Abstract

The sustainable development of modern aquaculture must rely on a significant reduction of the fish meal (FM) used in aquafeed formulations. However, FM substitution with alternative ingredients in diets for carnivorous fish species often showed reduced nutrient absorption, significantly perturbed metabolisms, and histological changes at both hepatic and intestinal levels. In the present study, rainbow trout (*Oncorhynchus mykiss*) were fed three different experimental aquafeeds. A control diet with higher FM content (27.3%) than two test formulations in which FM was substituted with two more sustainable and promising alternatives: insect meal (*Hermetia illucens* larvae = 10.1%, FM = 11.6%) and poultry by-products meal (PBM = 14.8%; FM = 11.7%). Combined metabolomics and proteomics analyses of fish liver, together with histological examination of liver and intestine demonstrated that a well-balanced formulation of nutrients in the three diets allowed high metabolic compatibility of either substitution, paving the way for a deeper understanding of the impact of novel raw materials for the fish feed industry. Results show that the main metabolic pathways of nutrient absorption and catabolism were essentially unaltered by alternative feed ingredients, and also histological alterations were negligible. It is demonstrated that the substitution of FM with sustainable alternatives does not have a negative impact on fish metabolism, as long as the nutritional requirements of rainbow trout are fulfilled.

## 1 Introduction

The valorization of finfish aquaculture in Europe heavily depends upon the ability of stakeholders within the supply chain to keep a high quality products while preserving aquaculture sustainable development at both environmental and economic levels. A well-known bottleneck of aquaculture development is the dependence of the aquafeed industry on wild fish-derived proteins and oils. To face this issue, a plethora of alternative protein sources are being tested as alternative fish feeds ingredients (Gatlin et al., 2007; Gasco et al., 2018; Cottrell et al., 2020). Among the most promising protein sources, certainly poultry by-products and insect larvae have played a chief role in recent investigations. Beyond being ideal candidates for a production approach based on a circular economy, poultry by-products meal (P) (Galkanda-Arachchige et al., 2020) and insect meal, in particular, *Hermetia illucens* larvae meals (I) (Gasco et al., 2021; Zarantoniello et al., 2021) allow the formulation of nutritionally balanced and highly digestible diets for the most largely farmed species in Europe (Galkanda-Arachchige et al., 2020; Gasco et al., 2021; Mohan et al., 2022). Although both I and P are being already included in commercial feed formulations for farmed finfish species, economic criticisms still represent a bottleneck to the massive use of insect meals (Arru et al., 2020), while issues related to consumer acceptance and regulatory restrictions so far slowed down the full exploitation of P (Zarantoniello et al., 2021).

Trout represent 75% of freshwater fish farmed in Europe, with over 250 thousand metric tons produced in 2019 (FAO, 2020). Early attempts of substituting fish meal (FM) with more sustainable vegetable alternatives in rainbow trout (*Oncorhynchus mykiss*) diets resulted in altered hepatic metabolism, reduced growth, intestinal inflammation and perturbation of gut microbiota (Desai et al., 2012). More recently, the use of processed animal by-products has attracted growing interest due to the suitable amino acid profile, high protein content and digestibility, and absence of antinutritional factors (Galkanda-Arachchige et al., 2020). For example, P has been successfully included in diets for rainbow trout, showing good digestibility, growth performances and survival, but leading to significant changes in fillet fat content, fatty acids composition and color (Parés-Sierra et al., 2014; Barreto-Curiel et al., 2016). According to the most recent literature (Galkanda-Arachchige et al., 2020; Mohan et al., 2022 and references therein) innovative experimental aquafeeds proposed for rainbow trout and exploiting P or I include at least 10% or, more commonly, higher percentages of FM. Dietary formulations with graded levels of P and I, used singly or in combination, to replace vegetable ingredients up to complete deprivation of FM, have been recently tested in rainbow trout (Dumas et al., 2018; Gaudioso et al., 2021; Randazzo et al., 2021), with mixed results. Some attempts to totally replace FM with P (Dumas et al., 2018) resulted in significantly lower growth and feed conversion and compromised protein and lipid deposition. Other studies, based on a combination of P and I in totally FM deprived diets, demonstrated no adverse effect on growth and inflammatory status but, on the contrary, showed significant ameliorative effects with respect to vegetable-rich diets (Gaudioso et al., 2021; Randazzo et al., 2021).

While a considerable effort has been so far spent on understanding the effects of innovative dietary formulations on zootechnical parameters and histology of several economically relevant aquaculture species, there is less information and data on their effects on the physiology of rainbow trout at molecular level. To more finely tailoring FM substitution in modern aquafeeds, however, species-specific strategies based on the deep knowledge of fish physiology should be optimized (Zarantoniello et al., 2021). Indeed, evaluation of fish feed performances are often mostly based on macroscopic indicators, such as growth, feed intake and conversion, and morphometric parameters, whereas microscopic investigations usually involve histology. Moreover, proximate and analytical composition of feed and fillet provide additional hints on feed digestibility and dietary effect. However, it has been already suggested that these approaches alone are not able to provide a complete picture of fish metabolism, wellness and health (Roques et al., 2020a). An insufficient understanding of the mechanistic origin of feed compatibility at molecular and metabolic levels is especially threatening if one accounts for the need, driven by sustainability reasons, to substitute FM and fish oil in modern aquafeed formulations.

The so-called “Omics” technologies represent an interesting evolution of biology which make possible an holistic investigation of living organisms, spanning across the study of the regulation of genes involved in protein, carbohydrate and lipid metabolisms towards the understanding of the actual adaptation of organisms at molecular level. By this approach, the response of a biological system to external stimuli (e.g., diet, rearing conditions, temperature) can be investigated at the level of genes, transcripts, proteins and metabolites. While each of the branches of omics technologies suffers of some limitations (Roques et al., 2020b), the combination of two or more of them opens the way to a more complete picture of the actual impact of experimental conditions on the organism under study. Moreover, integrated approaches have not been widely explored in studies on novel ingredients for aquafeeds (Lulijwa et al., 2022). In other words, if knowing the regulation of genes involved in a challenge study on fish can provide a first important indication of the main metabolic pathways involved in the biological response, the effect of post-transcriptional modifications and the final activity of proteins is uncertain. Similarly, if knowing the expression level of hepatic proteins in fish can give a detailed idea of active hepatic functions, only further characterization of small molecular weight metabolites, substrates or products of enzymatic activities, provides a more detailed and actual picture of the metabolic status of fish.

Hepatic proteome and metabolome reflect fish wellness and can be assumed as indicators of stress conditions (Carrera et al., 2020). Proteomic investigations on FM replacement in rainbow trout suggested the strict relationship existing between changes in differential expression of liver proteins and metabolic consequences in farmed fish (Martin et al., 2003). In this regard, a recent study explained how dietary inclusion of I may have effects on the hepatic metabolism of methionine (Terova et al., 2020). It has been also recently suggested that the cellular stress and other observed alterations in the hepatic proteome of rainbow trout fed *Tenebrio molitor* larvae substituted diets appear to be proportional to diet composition and quality of dietary ingredients (Mente et al., 2022). Moreover, it has been shown by means of Nuclear Magnetic Resonance (NMR)-based metabolomics approaches how a vegetable diet progressively substituted with *H. illucens* larvae hydrolysate affected both growth and metabolism of rainbow trout (Roques et al., 2020a).

Omics technologies can be integrated with more conventional analyses, although results may not necessarily correlate with each other as a function of diet composition. For example, a recent metabolomics work on modern diets for rainbow trout devoid of FM but containing graded levels of *Hermetia illucens* hydrolysate, led to a significant perturbation of underlying fish physiology but nevertheless resulted in an overall good zootechnical performance (Roques et al., 2020a). Similarly, since the effect of FM substitution at the intestinal level is attracting an ever growing interest, comparing histology and other physiological results might shine light on nutrient absorption and metabolism. Early studies evaluated the sensibly altered intestinal histology and nowadays, the focus is on microbiota at the gut level, which is thought to critically modulate trout health and wellness (Terova et al., 2019; Gaudioso et al., 2021; Rimoldi et al., 2021; Terova et al., 2021).

In the present work, we discuss a combined metabolomics and proteomics investigation on the effect of FM substitution with more sustainable alternative ingredients, P and I, on the physiology of rainbow trout. To further investigate how such dietary substitution affects liver, intestine and muscle tissues, we compare the derived metabolic picture with evidences from histology of liver and intestine and with the lipid fingerprints of fillets.

## 2 Materials and Methods

### 2.1 Experimental Design

Farmed rainbow trout were grown at Società Agricola Fattorie del Pesce, Cerano (NO), Italy (authorization of the Italian Ministry of Health Nr. 143811 granted on 19/03/2019). Fish were fed three extruded commercial isonitrogenous, isolipidic, and isoenergetic diets (Supplementary Material 1 in Table 1). In particular, a control diet (C) with higher FM content (27.3%) than two test formulations in which FM was substituted with two alternatives were tested. In the test diets, FM was replaced with either insect meal (I: *Hermetia illucens* meal = 10.1%, FM = 11.6%) or poultry by-products meal (*P* = 14.8%; FM = 11.7%) (Supplementary Material 2 in Table 1). At the beginning of the trial, 322 fish/tank (individual average weight: 156 g ± 0.003) were weighted and divided in 12 tanks. Therefore, each diet was allocated to four tanks containing 50 kg ± 0.206 g of rainbow trout. Fish were fed twice a day a ration of 0,8–1% of the tank biomass, 7 days a week. Ration was adjusted as a function of fish growth during the trial.

**FIGURE 1:**
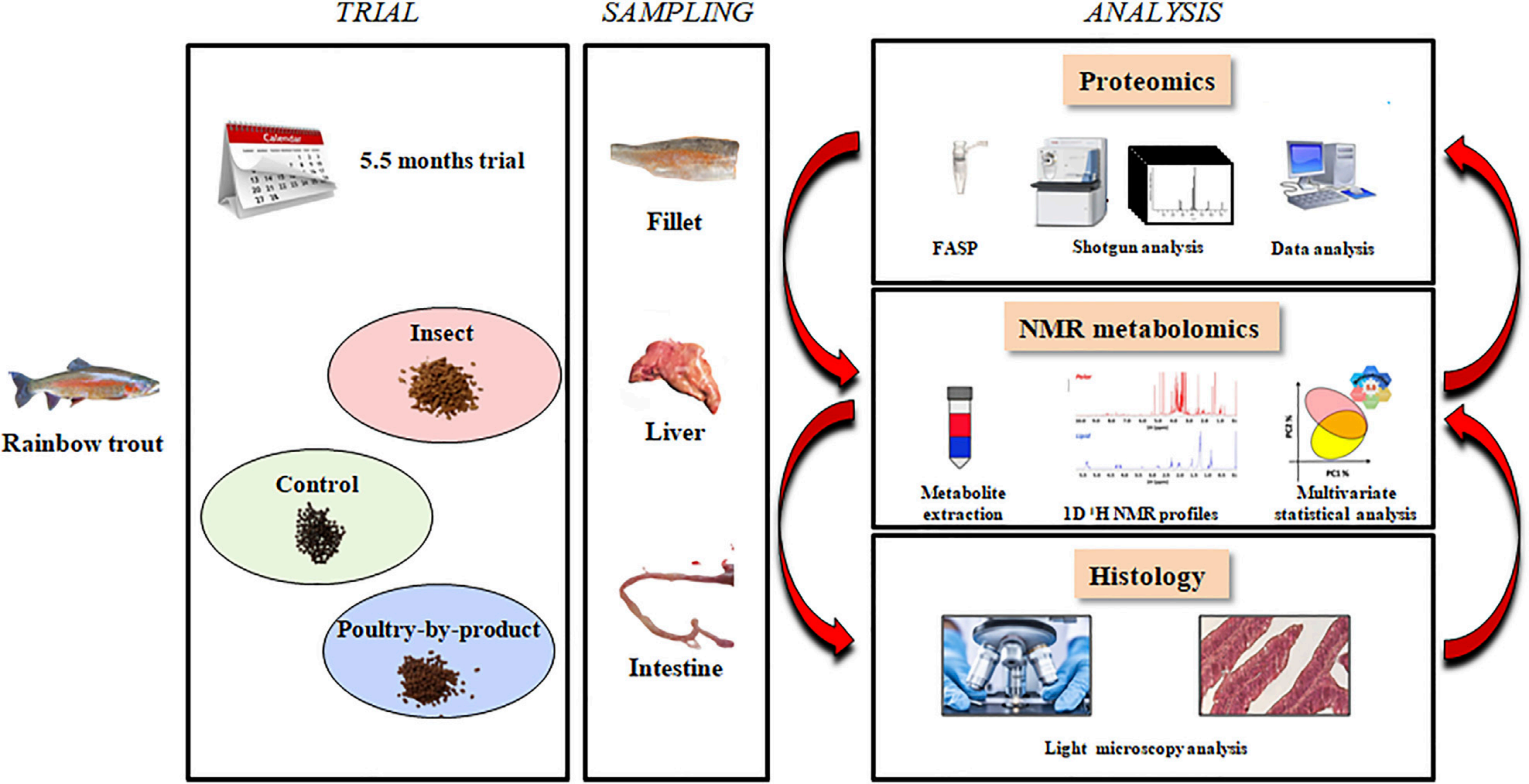
Workflow in the proteomics, metabolomics and histology experiments.

At the end of the trial (162 days), fish were sacrificed by over anaesthesia (MS-222, PHARMAQ Ltd., Fordingbridge, Hampshire, United Kingdom; 500 mg/L) and then severing the spinal cord. Proteomic analysis was performed on liver samples (two fish from each of three tanks for each diet), the metabolomic analysis on liver and muscle samples (10 fish from three tanks for each diet), the histological analysis on liver and intestinal (distal and proximal) samples (two fish from four tanks for each diet) (Figure 1).

### 2.2 Liver Proteomics

#### 2.2.1 Protein Extraction and Quantification

For each group, six liver samples (two from three tanks) were subjected to protein extraction according to Ghisaura et al. (2019) with some modifications. Briefly, a small portion of each tissue (200 mg) was placed in a 2 ml Eppendorf safe-lock tube (Eppendorf, Hamburg, Germany) and immersed in lysis buffer (0.065 M Tris-HCl pH 6.8, 1% dithiothreitol, 2% sodium dodecyl sulfate) in 1:4 (w/v) ratio, plus protease inhibitor cocktail (Protease Inhibitor Cocktail for General Use, Sigma-Aldrich, Saint Louis, MO, United States) as indicated in the manufacturer instructions. Samples were then subjected to three cycles of bead beating for 5 min at 30 oscillations/s in a TissueLyser mechanical homogenizer (Qiagen, Hilden, Germany), and frozen between homogenization cycles to facilitate tissue disruption and prevent excessive sample heating. Samples were then centrifuged for 15 min at 14,000 × *g* at 4°C. The final supernatants were collected as the liver protein extracts, diluted 1:50 in lysis buffer and quantified with the Pierce 660 nm Protein Assay Kit (Thermo Scientific - Rockford, IL, United States). Protein extracts were stored at −80°C until use.

#### 2.2.2 One-Dimensional Electrophoresis

Protein profiles were qualitatively evaluated by Sodium Dodecyl Sulphate-PolyAcrylamide Gel Electrophoresis (SDS-PAGE). Aliquots of 10 µg of each extracted protein mixture were incubated with a reducing Laemmli buffer (Laemmli, 1970) and subjected to SDS-PAGE on Any kD™ precast polyacrylamide gels (BioRad, Hercules, CA, United States). Coomassie SimplyBlue Safe Stain (Invitrogen, Waltham, MA, United States) was used for visualizing protein bands and an ImageScanner III (GE Healthcare, Little Chalfont, United Kingdom) to digitalize the gels.

#### 2.2.3 Shotgun Proteomics Analysis

Samples were also investigated by shotgun proteomics analysis. More in detail, 100 µg of protein extracts were subjected to on-filter reduction, alkylation, and trypsin digestion according to the filter-aided sample preparation protocol (Wiśniewski et al., 2009), with minor modifications (Tanca et al., 2013; Ghisaura et al., 2016) using Amicon Ultra-0.5 centrifugal filter units with molecular weight cut-off of 10 kDa (Millipore, Billerica, MA, United States). After digestion, peptide concentration was extrapolated by measuring the absorbance at 280 nm with Nanodrop 2000 (Thermo Fisher Scientific, Waltham, MS, United States) spectrophotometer and building a calibration curve with the MassPREP *E. Coli* Digest Standard (Waters, Milford, MA, United States) (Pisanu et al., 2018).

Protein digests were analyzed by Liquid Chromatography-tandem Mass Spectrometry on an LTQ-Orbitrap Velos instrument interfaced with an UltiMate 3000 RSLCnanoLC system (Thermo Fisher Scientific), as described previously (Abbondio et al., 2019). Specifically, 4 µg of each peptide mixture were concentrated onto a trapping precolumn (Acclaim PepMap C18, 75 μm × 2 cm nanoViper, 3 μm, 100 Å, Thermo Fisher Scientific) and separated on a C18 RP column (Acclaim PepMap RSLC C18, 75 μm × 50 cm nanoViper, 2 μm, 100 Å, Thermo Fisher Scientific) at a flow rate of 250 nl/min using a linear gradient of 245 min from 5 to 37.5% of eluent B (0.1% formic acid in 80% acetonitrile) in eluent A (0.1% formic acid). Full-scan was performed in the Orbitrap with a resolution of 60,000 at 445.12 m/z, and the 10 most intense ions of every scan were selected and fragmented. Higher Energy Collisional Dissociation, performed at the far side of the C-trap, was used as fragmentation method by applying a 35% value for normalized collision energy, MS/MS resolution of 7,500, an isolation width of m/z 3.0, a Q-value of 0.25, and an activation time of 0.1 ms. Nitrogen was used as collision gas.

#### 2.2.4 Peptide Identification

Peptide identification was carried out using Sequest-HT as search engine and Percolator for peptide validation False Discovery Rate (FDR) < 1% within the Proteome Discoverer (v. 2.4.1.15 Thermo Fisher Scientific) software. Protein and peptide identifications were obtained according to Palomba et al. (2021b) with minor modifications and using as database protein sequences belonging to the genus *Oncorhynchus*, downloaded from UniProt website (The UniProt Consortium, 2021) (https://www.uniprot.org/) on January 2021. Protein and peptide quantification was performed by an MS1-based label-free approach, considering the integrated peak area of the most abundant peak at the apex of the chromatographic profile (Palomba et al., 2021a). Functional characterization was achieved according to UniProt protein families and KEGG ortholog group classification (Kanehisa et al., 2021). Protein families were obtained through the UniProtKB accession numbers via the “retrieve” tool of the UniProt website (https://www.uniprot.org). KEGG orthologs information was achieved using the eggnog-mapper package (v.2.0.1) (Huerta-Cepas et al., 2016; Huerta-Cepas et al., 2017) available in a Galaxy server (https://proteomics.usegalaxy.eu) (Jagtap et al., 2015), according to the following parameters: eggnog database v.5.0.2; min e-value threshold 0.001.

#### 2.2.5 Differential Analysis

Differential analysis of proteomic data was performed comparing C vs*.* I, C vs*.* P and I vs*.* P groups. The Perseus computational platform (v.1.6.15.0) (Tyanova et al., 2016) was used considering as inputs peptide area values (aggregated based on the functional annotation levels: protein, protein family and KEGG ortholog), according to Palomba et al. (2021b). Briefly, features with missing values in more than three samples in at least one group were filtered out. Differential protein abundances between groups were tested with a two-tail Student’s *t*-test corrected by FDR based on permutation-adjustment, considering q = 0.05 as the threshold of significance. Additionally, unsupervised multivariate inspection by means of Principal Components Analysis (PCA) was also carried out on quantitative data of proteins, protein families and KEGG orthologs (Supplementary Material 3 in Table 2, Supplementary Material 4 in Table 3 and Supplementary Material 5 in Table 4). For this purpose, proteomic datasets were uploaded into the Statistical Analysis panel integrated into MetaboAnalyst 5.0 (http://www.metaboanalyst.ca) (Xia et al., 2009; Pang et al., 2021) and preliminary processed by means of normal sum, log transformation and Pareto scaling. In particular, PCA scores plots represent the coordinates of each acquired proteomic sample in the lower-dimensional space defined by the first two principal components (PC1 and PC2).

### 2.3 Liver and Muscle Metabolomics

#### 2.3.1 NMR Sample Preparation and Acquisition

Frozen liver and fish muscle portions derived from ninety specimens (i.e. 30 fish/diet) were used for NMR-based metabolomics purposes. Liver and muscle tissue extracts were prepared as previously described (Melis et al., 2017) and detailed in the Supplementary Material 6 in Table 5. One-dimensional (1D) ^1^H NMR spectra were acquired on a 600 MHz spectrometer (Bruker Biospin, Germany) at T = 298 K. Liver polar extracts were acquired using a simple pulse-acquisition sequence with suppression of residual water signal during relaxation delay and with optimized parameters for acquisition (p1 = 9.3 μs; number of scans ns = 64; relaxation delay d1 = 4.8 s; acquisition time AQ = 3.1 s). Lipid extracts from liver and fillets were acquired using a single pulse - acquisition - relaxation delay sequence. In particular, for liver extracts p1 = 9.65 μs; ns = 48; d1 = 6.0 s; AQ = 4.80 s; for fillets: p1 = 9.8 μs; ns = 32; d1 = 10.0 s; AQ = 3 s. NMR assignment of liver and fillet muscle polar and lipids metabolites was carried out based on previous published studies (Kullgren et al., 2010; Melis and Anedda, 2014), by two-dimensional correlations and by comparing reference spectra from Human Metabolome Database (HMDB) (http://www.hmdb.ca) (Supplementary Material 7 in Table 6, Supplementary Material 8 in Table 6, Supplementary Material 9 in Table 7 and Supplementary Material 10 in Table 7).

#### 2.3.2 NMR Data Processing and Statistical Analysis

Raw 1D ^1^H NMR spectra were calibrated against the chemical shift of the internal standard (TMSP) at 0 ppm, baseline corrected, aligned, and variable-size bucketed by using NMRProcFlow software (Jacob et al., 2017). To check the accuracy of NMR bucketing, signal to noise ratio (S/N) matrices were also generated using NMRProcFlow and average S/N was calculated for each bucket. Only metabolites which showed S/N higher than the lower limit of quantification (LLOQ) of S/N > 10 were taken into account (See Supplementary Material 8 in Table 6 for polar compounds and Supplementary Material 10 in Table 7 for lipids), in agreement with official recommendations (Sumner et al., 2007). Exported ^1^H NMR buckets data were then uploaded into the Statistical Analysis panel integrated into MetaboAnalyst 5.0 (http://www.metaboanalyst.ca) for multivariate data analysis inspection (Xia et al., 2009; Pang et al., 2021). PCA was used as unsupervised exploratory analysis to detect potential group clustering. As described above for proteomic data, ^1^H NMR buckets data were then subject to PCA by means of MetaboAnalyst 5.0 tool. Same data processing described in 2.2.5 (normal sum, log transformation and Pareto scaling) was adopted prior to generate PCA score plots for NMR-based metabolomics data.

### 2.4 Histology of Liver and Intestine

At the end of the feeding trial, two fish per tank (8 fish/diet) were anesthetized and then sacrificed as reported in 2.1. The intestine, divided into proximal and distal portions, and liver from each fish were dissected out and immersed into 10% Neutral Buffered Formalin (NBF) solution for fixation. Subsequently, samples were embedded in paraffin following standard histological protocol. Sections of 5 μm were cut using a microtome (Leica RM2245) and stained with hematoxylin and eosin (H&E) for light-microscope analysis. The obtained slides were then examined under light microscope (Zeiss Axiophot Microscope), photographed with digital camera (CMOS Discovery C30), and processed using Fiji software (open-source Java-based imaging program). In order to identify abnormalities and tissue alterations, two different evaluation systems were used for the two target organs. For the intestine, the protocol described by (Escaffre et al., 2007), was adopted. In particular, the following morphological parameters were measured: villi height (ViH); villi width (ViW), and the submucosal layer thickness (SMT). The data obtained were tested for normality distribution and then, in order to identify differences between groups, it was performed the one-way ANOVA analyses followed by Dunn’s post hoc test for multiple comparisons, using Past3 software (Hammer et al., 2001). Statistical significance was set at *p* < 0.05. For the morphological analysis of liver, a semi-quantitative scoring system was used, in accordance with (Rodrigues et al., 2017). In particular, for each section, four different histopathological changes were considered: nuclear displacement (ND), hepatocytes vacuolization (HV), irregular nuclei shapes (NS), and cellular hypertrophy (CH) (Caballero et al., 2004; Camargo and Martinez, 2007). The obtained values were chosen using a grading scale of the phenomenon (1 = not observed/few, 2 = medium, 3 = severe).

### 2.5 Integrated Metabolomic/Proteomic Liver Pathway Analysis

Reconstruction of metabolic pathways occurring in liver trout was performed by combining all unambiguous identified metabolites and proteins. For this purpose, integrated metabolomics and proteomics analysis was carried out by means of the MetaboAnalyst panel “Joint Pathway Analysis”. by using KEGG ID and KEGG orthologs as input entries (Supplementary Material 11 in Table 8 and Supplementary Material 12 in Table 9). Functional metabolic pathways were enriched by means of the Kyoto Encyclopedia of Genes and Genomes (KEGG) database (http://geneontology.org/), using zebrafish (*Danio rerio*) as model organism. Identified pathways were ranked in order of importance by considering the percentage of matched data uploaded (Hits) with respect to the total number of metabolites and proteins in each pathway (Total). Moreover, in order to evidence the main metabolic pathways, FDR < 0.05 and Hits > 5 were adopted as threshold limit parameters.

## 3 Results

### 3.1 Proteomics

In this study the analysis of protein extracts from the liver of rainbow trout (*Oncorhynchus mykiss*), using a label-free shotgun approach, allowed to identify and quantify a mean of 29,814 ± 1,326 peptides sequences (32,817 in total, see Table 1 and Supplementary Material 13 in Table 10 for details) belonging to a mean of 4,222 ± 111 proteins (4,472 in total). Protein identification and quantification data are reported more in detail in Supplementary Material 3 in Table 2 and Supplementary Material 14 in Table 11. The number of proteins identified and quantified in this study was closely comparable to those recently reported in two research articles by Causey et al. (2018) and Farinha et al. (2021) for the liver of rainbow trout and European seabass (*Dicentrarchus labrax*), respectively. In addition, after functional annotation, we found a mean of 2,042 ± 46 KEGG orthologs and 1,057 ± 20 protein families related to this identification (the complete list of KEGG orthologs and protein families identified and quantified are reported in Supplementary Material 4 in Table 3 and Supplementary Material 5 in Table 4, respectively).

**TABLE 1:** Shotgun proteomics analysis metrics. SD, standard deviation; CV, coefficient of variation; C, control diet; I, insect meal diet; P, poultry by-products meal diet.

Groups	Peptides		Proteins		KEGG orthologs		Protein families	
	Mean ± SD	CV (%)	Mean ± SD	CV (%)	Mean ± SD	CV (%)	Mean ± SD	CV (%)
All samples	29,814 ± 1,326	4.4	4,222 ± 111	2.6	2,042 ± 46	2.3	1,057 ± 20	1.9
C	30,008 ± 596	2.0	4,248 ± 54	1.3	2,054 ± 21	1.0	1,065 ± 9	0.8
I	29,257 ± 2,220	7.6	4,170 ± 184	4.4	2,021 ± 77	3.8	1,046 ± 32	3.2
P	30,177 ± 340	1.1	4,248 ± 18	0.4	2,051 ± 10	0.5	1,060 ± 3	0.3

#### 3.1.1 Differential Analysis

**FIGURE 2:**
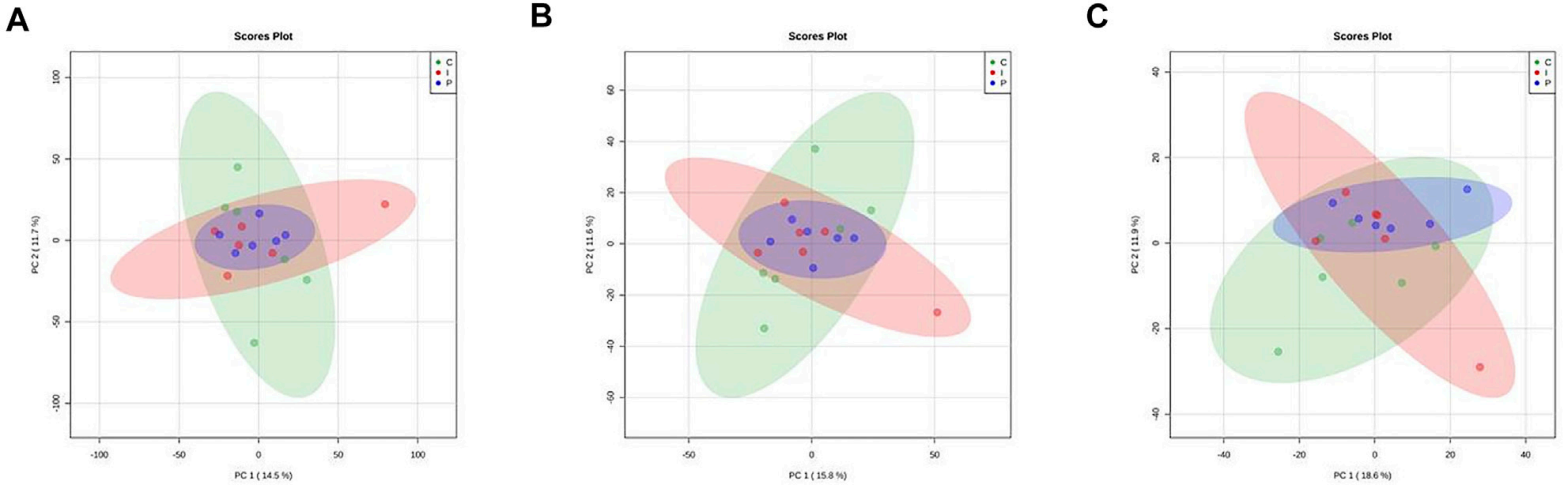
Principal component analysis (PCA) scores plots related to quantitative data of proteins **(A)** KEGG orthologs **(B)** and protein families **(C)** obtained from liver of rainbow trout subjected to different feed treatment (C: control diet, green; I: insect meal diet, red; P: poultry by-products meal diet, blue). Colored ellipses describe the T^2^-Hotelling 95% confidence intervals of each dietary group.

Exploratory unsupervised inspection by PCA (Figure 2) showed no discernible clustering according to feed treatments, using as input quantitative data of proteins, KEGG orthologs and protein families. Indeed, the first two PCs cover only from 14.5 to 18.6% of the model variability.

Then, we also compared the liver proteome of C, I and P fish groups with the aim of identifying differentially abundant proteins, protein families and KEGG orthologs that could be associated with diet. As a result, no protein, KEGG ortholog or protein family has been found to be statistically modified by dietary treatment (the results of differential analysis for each comparison are reported in Supplementary Material 15 in Table 12, Supplementary Material 16 in Table 13 and Supplementary Material 17 in Table 14. for protein, protein families and KEGG orthologs, respectively).

Among potential markers of lipid metabolism, glycerol-3-phosphate dehydrogenase, glyceraldehyde-3-phosphate dehydrogenase, several isoforms of fatty acid binding proteins, choline dehydrogenase, choline transporter-like protein, proteins of the carnitine/choline acetyltransferase family, dimethylglycine dehydrogenase, glycine cleavage system P protein, glycine N-methyltransferase, S-adenosylmethionine synthase and homocysteine-responsive ER-resident ubiquitin-like domain member 1 were identified and quantified, and their expression level was found to be uncorrelated with dietary treatment.

At hepatic level, we have also annotated the expression of the enzymes that catalyze the aminoacylation reaction in the first step of protein translation (aminoacyl-tRNA synthetases), with comparable results regardless of the dietary treatment.

In this work, we annotated several proteins that can be associated with oxidative stress, such as myosin light polypeptide 6, hemopexin, superoxide dismutase, thioredoxin-dependent peroxide reductase, and data evidenced that they were not differentially expressed in the different dietary groups. We have also characterized glutathione peroxidases, S-(hydroxymethyl)glutathione dehydrogenase, glutathione synthetase, ABC-type glutathione-S-conjugate transporter, glutathione transferase, glutathione reductase, glutathione-dependent dehydroascorbate reductase, microsomal glutathione S-transferase 3, S-formylglutathione hydrolase, lactoylglutathione lyase and evidenced no diet-related effect.

### 3.2 ^1^H NMR Metabolomics

**FIGURE 3:**
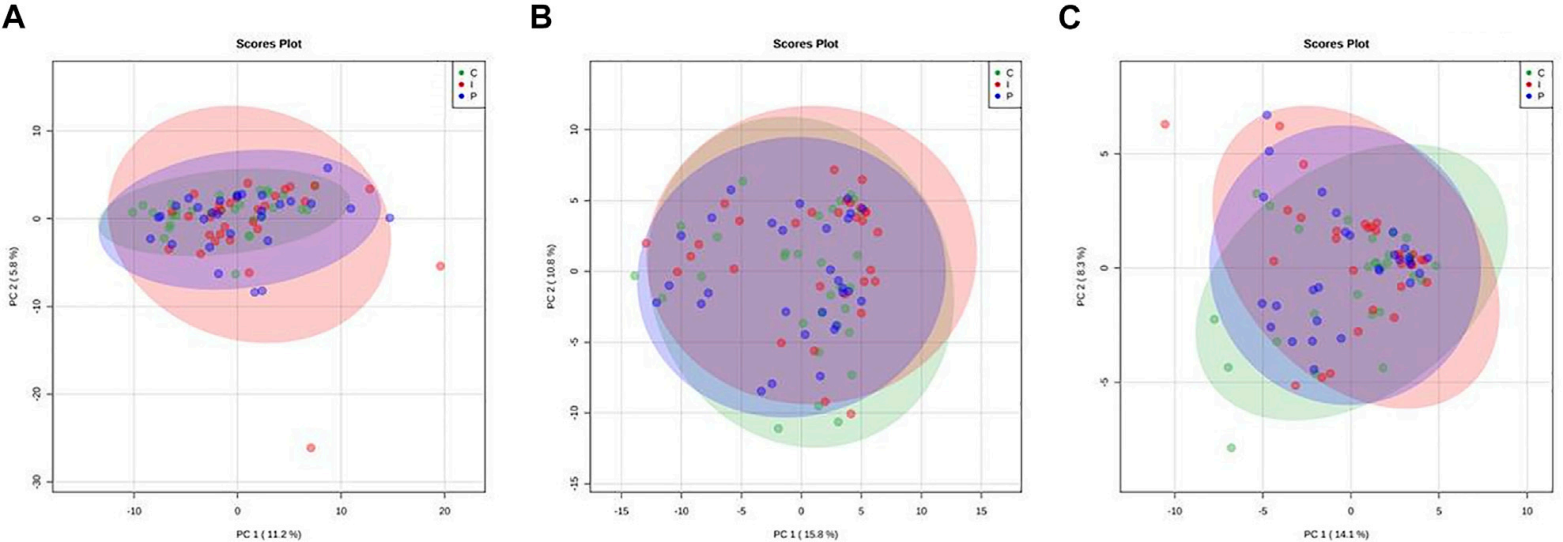
Principal component analysis (PCA) scores plots related to 1D ^1^H NMR spectra of liver aqueous **(A)** and lipid extracts **(B)** and from muscle lipid fraction **(C)** from rainbow trout fed different experimental diets (C: control diet, green; I: insect meal diet, red; P: poultry by-products meal diet, blue). Colored ellipses describe the T^2^-Hotelling 95% confidence intervals of each dietary group.

PCA score plots were also computed on ^1^H NMR data. Results from both trout liver and muscle metabolic profiles show an almost complete overlap of all dietary groups (Figure 3). On the axes are reported the percentages of the total variability of the first two main components (PC1 and PC2), which show that PCAs first components (PC1) cover only the 11–16% of the total variability in all models, thus suggesting that liver and muscle metabolite levels in all dietary groups are quite close to each other. Moreover, the analysis of NMR spectra of liver and muscle fillet confirms that no significant formation of primary and secondary oxidation products could be observed by the analysis of lipid extracts. This is quite informative of the oxidative status of trout tissues, since NMR is a very useful technique with respect to the more routine chromatographic characterization of lipid extracts in order to provide structural information of lipid oxidation products (Guillén and Goicoechea, 2007).

### 3.3 Integrated Hepatic Pathways Analysis

Through the KEGG-based integrated analysis of combined proteomic and metabolomic data (Supplementary Material 11 in Table 8 and Supplementary Material 12 in Table 9) a total of 121 pathways were initially annotated (Supplementary Material 18 in Table 15) and 30 of these were selected according to the ranking of Hits% and by overall filtering (FDR < 0.05 and Hits > 5). Moreover, we excluded two of them from the final list (carbon fixation in photosynthetic organisms and drug metabolism - other enzymes), since they appeared not relevant in the context of fish nutrition and metabolism. Selected pathways are listed in Table 2.

**TABLE 2:** Main metabolic pathways identified in trout liver as results of the integrated metabolomic and proteomic joint pathway analysis. Pathways are ranked considering hits percentage (Hits%) and filtered taking False Discovery Rate (FDR) < 0.05 and Hits > 5 as minimum thresholds.

Pathway	Total	Hits	Hits (%)	*p* val	FDR
Fatty acid metabolism	71	26	36.62	5.77E-11	2.58E-09
Valine, leucine and isoleucine degradation	105	36	34.29	1.06E-13	9.46E-12
Aminoacyl-tRNA biosynthesis	22	7	31.82	1.92E-03	1.27E-02
Ether lipid metabolism	29	9	31.03	5.39E-04	4.39E-03
Fatty acid elongation	51	15	29.41	1.64E-05	2.10E-04
Glutathione metabolism	76	22	28.95	2.38E-07	4.27E-06
Citrate cycle (TCA cycle)	78	22	28.21	3.94E-07	6.41E-06
Glycolysis/Gluconeogenesis	96	27	28.13	1.97E-08	5.03E-07
Alanine, aspartate and glutamate metabolism	98	27	27.55	3.18E-08	6.55E-07
Biosynthesis of unsaturated fatty acids	53	14	26.42	1.16E-04	1.09E-03
beta-Alanine metabolism	65	17	26.15	2.51E-05	2.81E-04
Fatty acid degradation	77	19	24.68	2.08E-05	2.48E-04
Glycine, serine and threonine metabolism	130	32	24.62	3.29E-08	6.55E-07
Various types of N-glycan biosynthesis	57	13	22.81	9.46E-04	7.06E-03
Glyoxylate and dicarboxylate metabolism	128	29	22.66	9.75E-07	1.45E-05
Histidine metabolism	62	13	20.97	2.15E-03	1.37E-02
N-Glycan biosynthesis	81	16	19.75	1.36E-03	9.34E-03
Tryptophan metabolism	117	23	19.66	1.41E-04	1.26E-03
Carbon metabolism	350	68	19.43	5.32E-11	2.58E-09
Biosynthesis of cofactors	360	68	18.89	1.98E-10	5.90E-09
Propanoate metabolism	113	21	18.58	6.04E-04	4.70E-03
Cysteine and methionine metabolism	151	27	17.88	2.03E-04	1.73E-03
Pyruvate metabolism	143	24	16.78	1.17E-03	8.34E-03
Biosynthesis of amino acids	235	38	16.17	1.05E-04	1.05E-03
Amino sugar and nucleotide sugar metabolism	174	25	14.37	7.92E-03	4.57E-02
Purine metabolism	274	37	13.50	4.10E-03	2.44E-02
Biosynthesis of secondary metabolites	1,460	187	12.81	1.76E-10	5.90E-09
Metabolic pathways	4,090	454	11.10	1.50E-38	2.69E-36

The selected 28 pathways, which represent the main metabolic routes active in rainbow trout liver and involved in the metabolisms of the tested diets, are summarized in Table 2. They mainly concern the metabolism of dietary amino acids, lipids and, to a minor extent, of carbohydrates. We detected a lower importance of metabolic routes related to this latter class of compounds in trout liver. Indeed, the lower ranking was observed (Table 2) for the energy metabolism of carbohydrates (glycolysis and gluconeogenesis) with respect to the metabolism of lipids and proteins, as well as regarding the interconversion of carbohydrates into amino acids (AA) through acetyl-CoA (citrate cycle or TCA cycle, pyruvate metabolism and amino sugars and nucleotide sugars).

**FIGURE 4:**
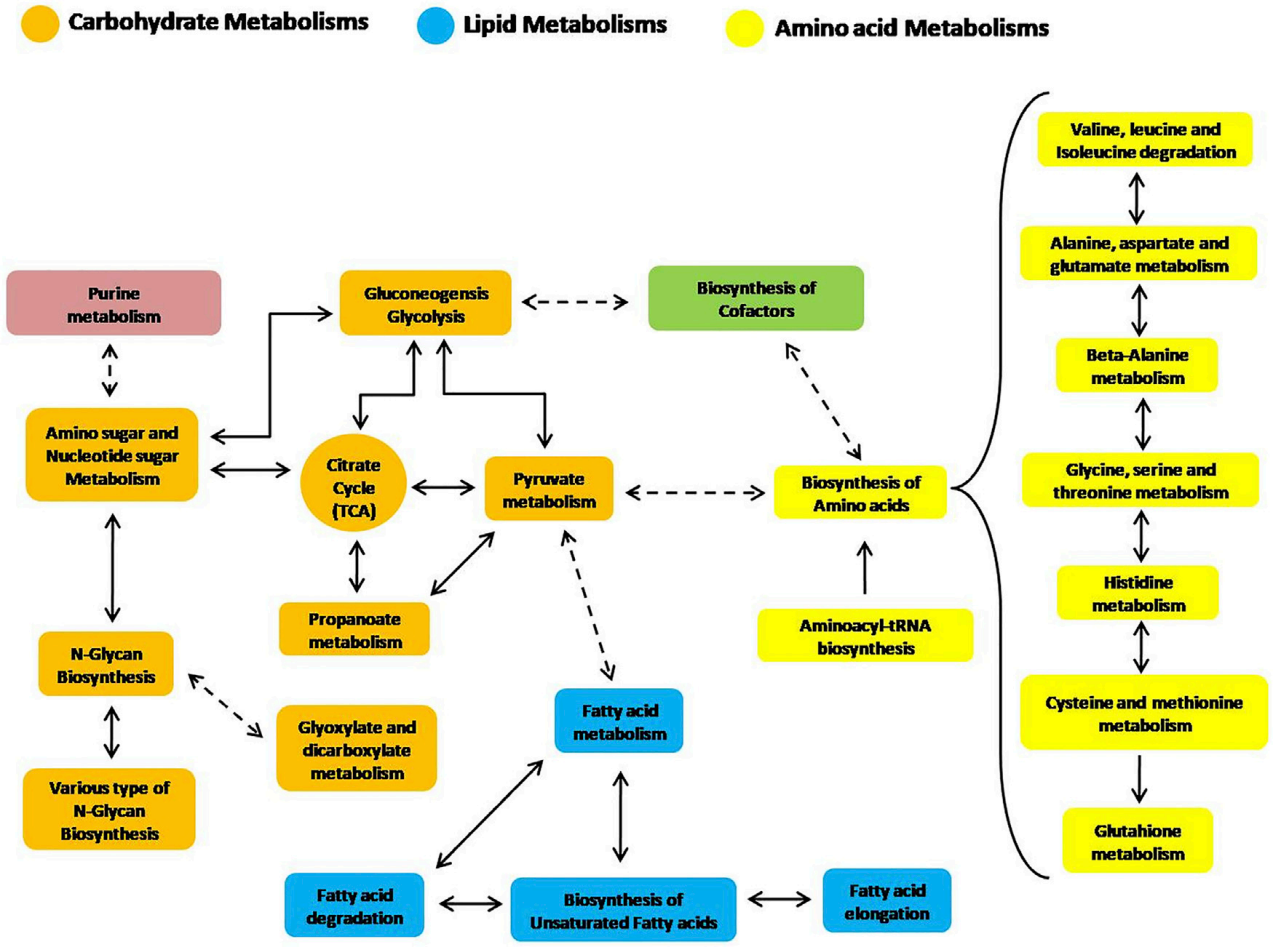
Reconstruction of significant liver metabolic pathways identified by an integrated analysis of proteomics and metabolomics data (based on the KEGG pathway module database).

Additionally, some other secondary or more general metabolic routes have also been reconstructed by the analysis of trout liver. The selected pathways derive from the combined examination of protein expression and metabolites levels detected in the hepatic tissue following consumption of the tested diets, therefore representing a comprehensive picture of the metabolic state of fish at the time of sampling. The intercorrelation of the aforementioned metabolic routes is schematically depicted in Figure 4 to guide the reader.

### 3.4 Histological Evaluation

**FIGURE 5:**
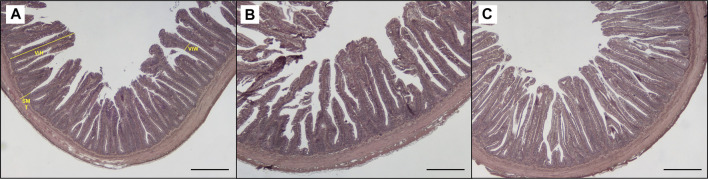
Light microscopy of proximal intestine portions of trout fed control diet **(A)**, poultry by products meal diet **(B)** and insect meal diet **(C)**. SMT, submucosal layer thickness; ViH, villi height; ViW, villi width. Hematoxylin and eosin, scale bar = 500 μm.

**FIGURE 6:**
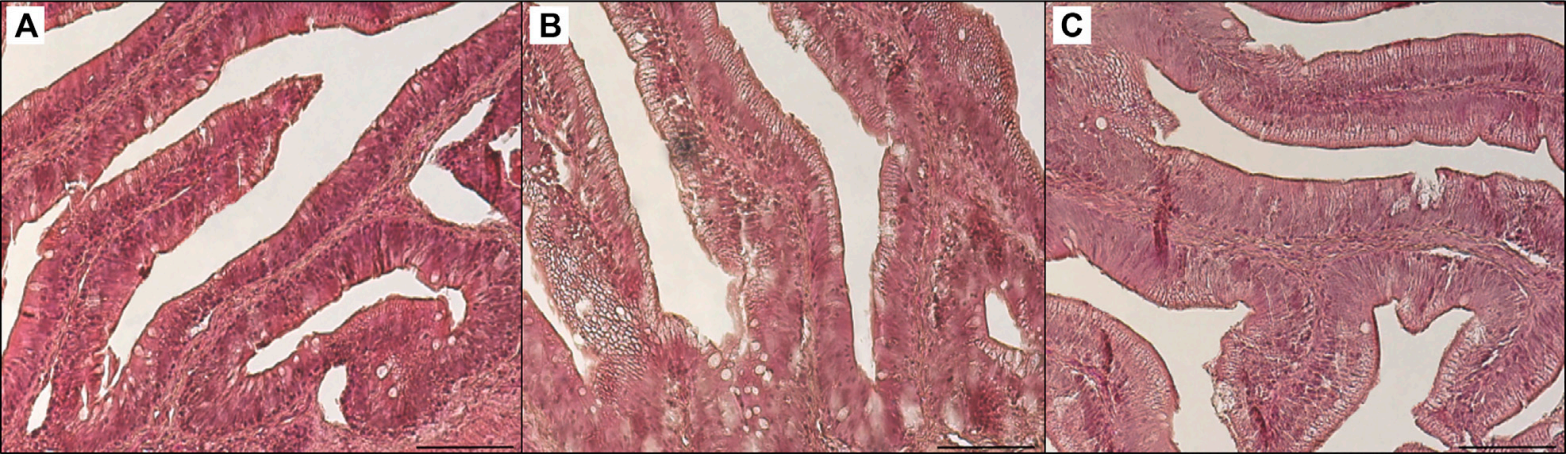
Light microscopy of distal intestine portions of trout fed control diet **(A)**, poultry by-products meal diet **(B)** and insect meal diet **(C)**. Hematoxylin and eosin, scale bar = 100 μm.

**FIGURE 7:**
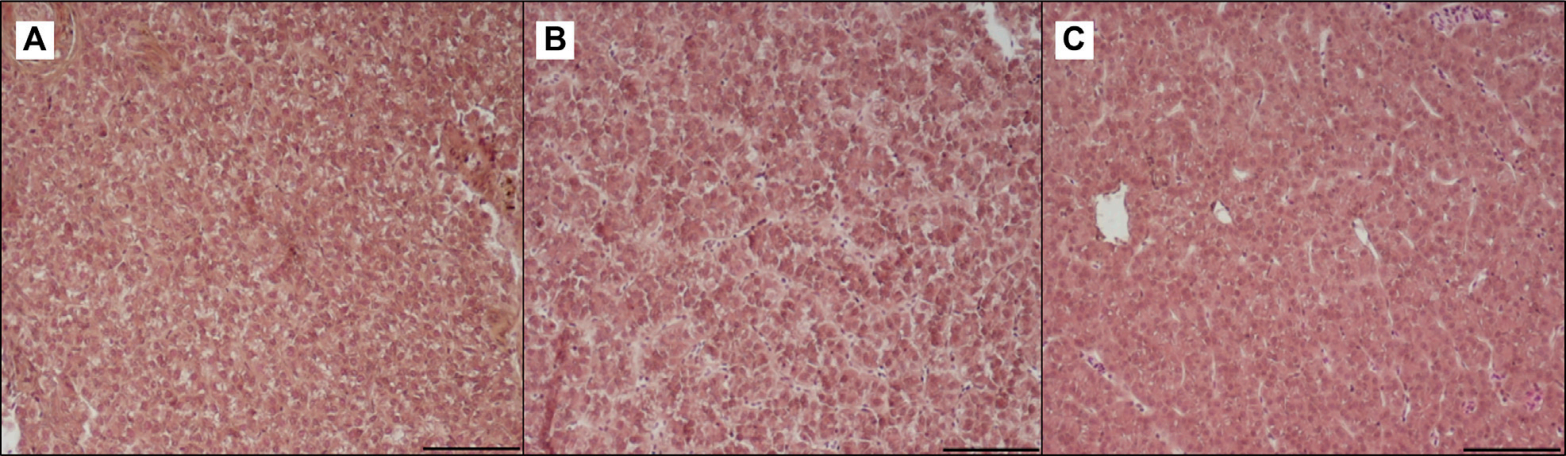
Light microscopy of liver of trout fed control diet **(A)**, poultry by-products meal diet **(B)** and insect meal diet **(C)**. Hematoxylin and eosin, scale bar = 100 μm.

Table 3 shows the data obtained from the measurement of the morphological parameters, divided by proximal and distal intestine, and liver in fish fed the C and the two experimental diets (I and P). The data showed that, regardless of the diet, no substantial alterations were present in the general morphology of the two analyzed organs. In the proximal and distal portions of the intestine, no significant differences were detected between the fish dietary groups for all the analyzed parameters (Figures 5, 6). In particular, the simple mucosal folds (ViH; ViW) appear well structured and numerous in the proximal intestine, whereas in the distal part, the simple folds were interspersed with many complex folds. For both intestinal portions, although the SMT seems larger, if compared with previous studies on rainbow trout (Randazzo et al., 2021), no significant differences, and no inflammation phenomena were detected between the dietary groups, suggesting a normal physiological condition of the organ structure. With regard to the hepatic tissue, all trout show a healthy liver with round central hepatocytes nuclei and only in rare cases, NS were detected, mostly in trout of the I dietary group. In comparison to the control group, fish fed I and P diets displayed slight vacuolization of the cells, located in some districts. However, this did not affect the general cell trophy and the physiological morphology of the organ (Figure 7).

**TABLE 3:** Histological changes in proximal and distal intestine and in the liver of rainbow trout fed different experimental diets. Proximal and distal intestine data are shown as mean ± standard error. Liver data are expressed as a grading scale (1 = not observed/few, 2 = medium, 3 = severe) (*n* = 4). ViH, villi height; ViW, villi width; SMT, submucosal layer thickness; ND, nuclear displacement; HV, hepatocytes vacuolization; NS, irregular nuclei shapes; CH, cellular hypertrophy. C, control diet; I, insect meal diet; P, poultry by-products meal diet.

Morphological parameters	Experimental diets		
Proximal Intestine	C	I	P
ViH (µm)	731.34 ± 25.11	735.74 ± 54.64	738.45 ± 34.65
ViW (µm)	91.08 ± 3.47	92.24 ± 4.34	91.66 ± 6.35
SMT (µm)	72.32 ± 3.30	74.58 ± 2.81	71.93 ± 2.03
Distal Intestine	C	I	P
ViH (µm)	740.89 ± 47.25	736.26 ± 68.23	737.60 ± 96.41
ViW (µm)	88.47 ± 3.18	86.63 ± 3.85	89.28 ± 7.19
SMT (µm)	58.26 ± 1.92	59.76 ± 5.04	57.36 ± 3.86
Liver	C	I	P
ND	1	1	1
HV	1	1–2	1–2
NS	1	1–2	1
CH	1	1	1

## 4 Discussion

### 4.1 The Methodological Approach

We discuss here the results of a combination of metabolomics and proteomics studies on rainbow trout liver and fillet, together with an integrated analysis of multi-platform experimental data, to better clarify the effect of modern aquafeed formulations in which FM was substituted with more sustainable alternatives, namely insect meal from *Hermetia illucens* and poultry by-products meal. The results from omics approaches are also discussed in light of histological evidences on liver and intestine, and exploiting specific and comprehensive databases to expand knowledge from protein expression and effective metabolism in farmed fish. According to the most recent findings, systematically reviewed in Mohan et al. (2022), new experimental diets intended for rainbow trout and exploiting *Hermetia illucens* meal as an innovative ingredient, are generally formulated including a minimum of approximately 10% of FM or higher percentages. To our knowledge, only few pioneering studies aimed at characterizing the effects of total deprivation of FM in rainbow trout diets (Dumas et al., 2018; Roques et al., 2020a; Gaudioso et al., 2021; Randazzo et al., 2021) and most of these studies did not characterize the specific molecular pathways involved in nutrient absorption and metabolism. As for FM replacement with poultry by-products meal, rainbow trout as many other freshwater fish species showed good tolerance of total removal of FM in aquafeeds (Galkanda-Arachchige et al., 2020), but again the knowledge of the metabolic fate of the corresponding nutrients at physiological level is rather scarce.

Discovery proteomics plays a key role in the study of a given proteome and the identification of valuable biomarkers (Parker and Borchers, 2014). For a long time, the gel-based approach, above all two-dimensional gel electrophoresis (2-DE), has been primarily employed as the favorite technique for the separation of proteins, but in the last decade gel-free approaches, also known as shotgun proteomics, have become more popular. Shotgun proteomics approach provides a higher proteome coverage. It is less affected by the typical limitations of gel-based studies (Chevalier, 2010), such as analysis of membrane, low-abundance and high molecular weight proteins, especially when 2D separation is involved (Westermeier and Scheibe, 2008), so that additional or complementary information can be obtained with respect to previous studies. Also in the aquaculture field, proteomics techniques are considered a robust methodology to evaluate quality and safety, as reviewed by Carrera et al. (2020). Proteomics holds the potential to provide useful information to improve the productivity, welfare, nutritional value and health of farmed fish and consequently it has become of great importance for the evaluation of farming conditions in aquaculture, including different aspects of dietary management, fish welfare and response to stress, food safety, and antibiotic resistance (Carrera et al., 2020).

NMR-based metabolomics provides a clear picture of metabolic substrates and products of metabolism of the fish subjected to different rearing conditions, and allows to define molecular details underlying the maintenance or impairment of the metabolic status after long-term consumption of the tested diets. State-of the art applications of this method to the field of fish nutrition has been reviewed (Alfaro and Young, 2018; Roques et al., 2020b; Lulijwa et al., 2022), with respect to which we believe the present study represents a step forward towards a more comprehensive understanding of dietary modulation of rainbow trout metabolism and, more generally, towards the optimization of more suitable multidisciplinary investigations of the impact of aquaculture practices on fish quality, health and welfare. In particular, as pointed out in a recent review (Lulijwa et al., 2022), studies on salmonids metabolism exploiting more than one single analytical platform are rather rare, and none of the reviewed works addressed the problem with more than two approaches.

In several previous scientific contributions, we provided evidence of the suitability of omics methods in the understanding of the metabolic effects of rearing conditions in fish. We evaluated liver protein profile using shotgun proteomics workflow to investigate the impact of feed formulations (Ghisaura et al., 2014) and cold stress (Ghisaura et al., 2019) on the metabolism of farmed gilthead sea bream (*Sparus aurata*, L.). Moreover, we have given evidence of the NMR-based metabolomics classification of *Sparus aurata* fed plant-based diets in different rearing conditions (Melis and Anedda, 2014) and we demonstrated the power of this approach in describing the molecular details of the sensitivity of gilthead sea bream to cold water temperature, and described the actual metabolic impairment caused by this stressful condition (Melis et al., 2017). We also demonstrated that fast and reproducible NMR fingerprinting methods are able to differentiate wild and farmed sea breams, and that the analysis is able to highlight the metabolic differences associated to different rearing sites (Melis et al., 2014). It is worth pointing out in this regard that previous evidences strongly suggest that our optimized NMR-based metabolomics and shotgun proteomics protocols are capable to easily highlight molecular differences between different groups of treatments even when each of these analytical methods is applied alone (Ghisaura et al., 2014; Melis and Anedda, 2014; Melis et al., 2014; Melis et al., 2017; Ghisaura et al., 2019; Roques et al., 2020a; Carrera et al., 2020; Lulijwa et al., 2022), so that the combination of both of them would likely perform much better in identifying differential molecular profiles, if any were present.

### 4.2 Dietary Modulation of Trout Metabolism

Figure 4 and especially Table 2 clearly suggest that the main metabolic pathways highlighted in rainbow trout liver, as supported by this study, are related to lipids and AA, thus reflecting the role of such metabolites as primary energy substrates in carnivorous fish with respect to carbohydrates (Polakof et al., 2012).

Moreover, it is worth recalling here that the main result obtained by the analysis of hepatic metabolism in trout fed the diets with different percentages of FM, was to show that modern dietary formulations with reduced FM content can be very well accepted by carnivorous fish like trout. The compatibility of these experimental feeds, beyond their effect on growth parameters and histological alterations, lead to a scarce repercussion of the substitution at physiological level, protein expression level and small metabolites catabolism and turnover. This result is very well explained by Figures 2 and 3, which clearly show that a metabolic effect of the substitution could be evidenced by neither proteomic nor metabolomic analytical approaches. This in turn implies that dietary modulation of trout metabolism at hepatic level has not been significantly affected by diet composition, and conversely all aquafeeds tested had the same impact on the capacity of rainbow trout to absorb and catabolize nutrients from the dietary sources provided. This is reasonably due to both the well balanced nutrient supply from the diets and the high quality of raw materials and extrusion process used. It is worth noting that a comparison with other studies performed by means of very similar analytical procedures (Lulijwa et al., 2022) shows the efficacy of the proposed omic approach in identifying any possible metabolic perturbation driven by dietary stimuli in aquatic species.

#### 4.2.1 Lipid Metabolism

Lipids and their constituent fatty acids are, along with proteins, the major organic constituents of fish, and thus play a fundamental role as sources of metabolic energy for these organisms (Tocher, 2003; Anedda et al., 2013). Moreover, lipid profile of fish is expected to significantly characterize key quality aspects of the edible part, and plays therefore a critical role in modulating the nutritional value and sensorial features of fillets (Kamal-Eldin, 2003).

Previous studies have highlighted some altered hepatic functions in fish involving lipid absorption, catabolism and transport. Metabolic changes in lipid biosynthesis and turnover were found to be driven by different stressful conditions, such as diet (Melis and Anedda, 2014; Casu et al., 2017), high stocking density, bacterial infections (Causey et al., 2018), exposure to chemicals and pollutants and other metabolically demanding situations (Melis et al., 2017; Zhu et al., 2018; Ghisaura et al., 2019). Glycerol-3-phosphate, glycerol-3-phosphate dehydrogenase, glyceraldehyde-3-phosphate dehydrogenase, several isoforms of fatty acid binding proteins and choline (and derived forms) are among the most relevant biomarkers of lipid metabolism in fish (Roques et al., 2020b). In our investigation, metabolomics and proteomics evidenced no significant difference in these key metabolites after consumption of aquafeeds in which FM has been substituted with I and P alternatives. Choline and the metabolites directly related to its metabolism, such as N,N dimethylglycine, dimethylamine and phosphocholine, represent an informative mirror of the actual metabolic state of fish (Roques et al., 2020b). They were shown to be altered in wild fish fed manufactured diet or farmed fish reared with plant-based diets (Melis and Anedda, 2014; Maruhenda Egea et al., 2015; Casu et al., 2017). Alterations of choline in fish liver could account for the impairment of the capacity of liver to process and transport dietary lipids to blood and peripheral tissues, and could thus represent a warning signal of fatty liver and steatosis. This was very clearly evidenced in other fish species (Melis et al., 2017; Ghisaura et al., 2019). However, in the present study, neither metabolite levels nor the expression of key proteins involved in choline metabolism (e.g., choline dehydrogenase, choline transporter-like protein, and several uncharacterized proteins of the carnitine/choline acetyltransferase family) and in the turnover of its derived metabolites such as dimethylglycine (dimethylglycine dehydrogenase, glycine cleavage system P protein, glycine N-methyltransferase), methionine (S-adenosylmethionine synthase) and homocysteine (homocysteine-responsive ER-resident ubiquitin-like domain member 1) were found to be significantly perturbed by FM substitution in feed formulations (Supplementary Material 15 in Table 12).

#### 4.2.2 Protein Metabolism

In previous works, protein catabolism and turnover were found to be significantly perturbed in rainbow trout liver following a plant-based dietary treatment (Martin et al., 2003). On the other hand, it was found that substitution of FM with different insect meals, including *Hermetia illucens*, in diets intended for European sea bass (Mastoraki et al., 2020), did not evidence significant changes in the amino acid profile of muscle fillets and proved good protein retention performances with respect to the high-FM diet fed fish. In the same work on sea bass, activities of liver enzymes involved in catabolizing amino acids (alanine aminotransferase, aspartate aminotransferase and glutamate dehydrogenase) were not affected by the replacement, thus suggesting an overall good compatibility of *Hermetia illucens* meals with carnivorous fish species. It is worth recalling (Barroso et al., 2014) that insect meals generally possess a well-balanced amino acid profile, and that of Diptera (e.g., *Hermetia illucens* larvae) is most similar to FM regarding essential and limiting amino acids content. Methionine content is close to that of FM, while histidine, lysine and threonine are more concentrated (Barroso et al., 2014).

Amino acids (AA) are known not only as building blocks for protein synthesis but also as primary energy substrates in carnivorous fish like trout. Indeed, AA liver deamination and transamination provide carbon backbones for gluconeogenesis and citrate (TCA) cycle and to fatty acid synthesis (Kaushik and Seiliez, 2010). The crucial role played by several metabolic pathways related to liver amino acid pool was highlighted in our work by integrated omic analysis. In previous works, AA pool impairments in fish tissues have been related to a corresponding dietary imbalance (such as deriving from scarcity in some AA derived from alternative fish feed ingredients (Wagner et al., 2014; Jasour et al., 2017). In particular, metabolism of branched chain AA (BCAA) seems to play a predominant role: increased dietary level of leucine and valine has been earlier related to TCA inhibition, because these two BCAA are preferentially transaminated to produce glutamate, pyruvate and alanine, that represent the entry points into the TCA cycle (Wagner et al., 2014). At the same time, a large supply of these AA from diet, as derived by replacement with dietary hydrolysates, has proven to stimulate their metabolism, thus decreasing the gluconeogenesis pathway. Also, BCAA catabolism plays an essential role in modulating protein synthesis as well as in the regulation of leptin and GLP-1 hormones release, thus influencing blood glucose concentration and consequently fish food intake (Ahmad et al., 2021). Furthermore, alanine and glycine, included in some highlighted liver pathways, are known to be important substrates of the TCA cycle and it was previously suggested they are used to counterbalance the energy-deficient state in fish fed plant-based diets (Li et al., 2009; Casu et al., 2017; Wei et al., 2017).

Among the most represented metabolic routes at hepatic level, enzymes that catalyze the aminoacylation reaction in the first step of protein translation (aminoacyl-tRNA synthetases), catalyze the ligation of tRNA to AA. SNP variations in the aminoacyl-tRNA biosynthesis have been previously associated with plant-based diets in zebrafish (Ulloa et al., 2015) and alteration of transcription regulation through aminoacyl-tRNA biosynthesis was also observed in juvenile rainbow trout fed vegetable oils (Panserat et al., 2008). The same biosynthetic pathway was found to be highly perturbed at the systems level, combining muscle and liver expressed genes, in selected vegetarian rainbow trout strains (Abernathy et al., 2017). This evidence supports the key role played by regulatory RNAs in fish growth and nutrient metabolism in fish, including rainbow trout and substantiates the evidence that AA biosynthesis and catabolism are among the most important hepatic pathways in juvenile rainbow trout. No diet-related perturbation of protein translation processes was observed in this study.

#### 4.2.3 Carbohydrate Metabolism

In agreement with the hypothesis that carbohydrates are a secondary and less compatible source of energy in carnivorous fish, biomarkers of carbohydrates metabolisms ranked after those of protein and lipids. TCA cycle can be considered as a key metabolic mechanism in fish energy homeostasis, as it is connected to the main metabolic pathways, especially through the supply of glucose and conversion of AA and lipids to provide ATP. High dietary carbohydrate levels, such as those derived from plant-based feeds, have proven to severely affect fish liver glycolysis and gluconeogenesis pathways. In particular, alterations of liver central metabolites such as d-glucose and also lactate provide evidences of the altered liver function and consequently fish muscle metabolism (Maruhenda Egea et al., 2015). These effects were not observed in this study. Moreover, lower glucose content in fish liver has been already associated with higher glycogen content and higher hepatosomatic index, as seen in both fasted rainbow trout and herbivorous species fed high-carbohydrate diets (Kullgren et al., 2010; Prathomya et al., 2017).

#### 4.2.4 Oxidative Stress

In living organisms subjected to stressful conditions, such as fish in some intensive farming environments, an imbalance is generated between the production of reactive oxygen species (ROS) and the natural antioxidant defense system of the organism itself, thus causing a cascade of molecular (metabolic) responses involving lipid peroxidation, DNA hydroxylation, proteolysis, protein misfolding and denaturation, immune dysfunction, cellular apoptosis and cell damage. Several biomarkers for such conditions have been identified so far in fish, both belonging to the classes of small molecular weight metabolites, enzymes and proteins: the presence of high amounts of lipid peroxides and total glutathione in liver, kidney and muscle, differentially enhanced expressions of superoxide dismutase, glutathione peroxidase, glutathione reductase, and glutathione S-transferase at hepatic level are generally assumed as metabolic signatures of oxidative stress in fish. Moreover, diet-related dysregulation of proteins involved in oxidative stress, such as myosin light polypeptides, peroxiredoxins and hemopexins have been observed following FM substitution with vegetable alternative ingredients in fish feeds (Carrera et al., 2020). Several of the aforementioned biomarkers were identified in our study but did not show associations with dietary groups. Myosin light polypeptide 6, hemopexin, superoxide dismutase, thioredoxin-dependent peroxide reductase, were previously associated with the antioxidant system of trout (Pacitti et al., 2014; Kurhaluk, 2019). Thioredoxin reductases are one of the main intracellular redox systems, being very important to regulate ROS accumulation. Other key functional molecules for regulating oxidative stress at hepatic level in fish are those involved in the glutathione metabolism. We have characterized glutathione peroxidases, S-(hydroxymethyl)glutathione dehydrogenase, glutathione synthetase, ABC-type glutathione-S-conjugate transporter, glutathione transferase, glutathione reductase, glutathione-dependent dehydroascorbate reductase, microsomal glutathione S-transferase 3, S-formylglutathione hydrolase, lactoylglutathione lyase without evidencing significant effect of diet in their expression levels.

#### 4.2.5 Histological Evidences

The present data demonstrated that the partial FM substitution with the two more sustainable and promising alternatives, such as I and P, did not affect the hepatic and intestinal structure of adult rainbow trout. As already demonstrated by several previous studies, P and I, used alone or in combination, can successfully replace FM in aquafeeds. Indeed, Randazzo et al. (2021), Bosi et al. (2021), and Elia et al. (2018), stated that moderate to high level of P and I do not affect the gross morphology of intestine and liver in trout and suggested that these ingredients can improve gut health and growth performances in fish fed with vegetable-rich diets. In line with those findings, following the present morphological analysis, no differences were detected between the two experimental groups as compared to the control. No inflammation signs in either hepatic parenchyma or the mucosal layer of the intestine, highlighting the potential of both tested sources of protein in the diet to be a beneficial and sustainable alternative to FM.

### 4.3 Effect of Fishmeal Replacement on the Lipid Profile of Fillet

As expected from the combined analysis of liver proteome and metabolome, the impact of the substitution of FM in the tested feeds on the lipid profiles of trout fillets was found to be negligible. Lipid metabolisms and quality profiles resulting from the feeding trials with I and P were in fact not significantly affected, thus resulting in a high-quality product that does not allow to discriminate a sustainable production from its high FM-fed counterpart. As it is mentioned above, formation of peroxides and ROS was not observed in the muscle of rainbow trout fed the three tested diets, meaning that oxidative stress did not significantly affect fish composition in the edible part.

## 5 Conclusion

By combining integrated proteomic and metabolomic inspection on liver functions with histology of liver and intestine, we demonstrated that two experimental diets, in which FM has been replaced for more than 50%, resulted in a very high metabolic compatibility for rainbow trout juveniles. The tested diets, in which FM was reduced to less than 12%, did not significantly affect nutrient metabolism with respect to a control diet with 27.3% of FM. Moreover, they did not show a significant negative impact on the lipid quality of fillets, nor on histology of liver and intestine.

The main and secondary metabolic routes of nutrient metabolisms have been described in detail, thus providing an useful guide for future research on the metabolic effect of sustainable alternative ingredients to FM in aquafeeds intended for this species. We suggest omic approaches are combined to more routine zootechnical, morphological, histological and economic investigations in future studies on new dietary ingredients. Moreover, the integrated omic approach presented here would likely be very informative to characterize the possible long-term effects of innovative ingredients on this farmed species, since metabolic perturbations are supposed to be more rapidly evidenced than the effects on fish growth and health, which would likely appear over longer timescales.

## Data Availability

The datasets presented in this study can be found in online repositories. The names of the repository/repositories and accession number(s) can be found below: http://proteomecentral.proteomexchange.org/, PXD033201.
