# Supplementary material for: On the Compatibility of Fish Meal Replacements in Aquafeeds for Rainbow Trout. A Combined Metabolomic, Proteomic and Histological Study
Source: Front Physiol. 2022 Jun 29;13:920289. doi: 10.3389/fphys.2022.920289 (PMC9276982; doi:10.3389/fphys.2022.920289)
Supplement: Supplementary file 4 [file Table1.DOCX]

| **COMPOSITION** | **C** | **I** | **P** |
| --- | --- | --- | --- |
| **Dry matter** | 92.8 | 93.4 | 92.3 |
| **Crude Protein** | 44.8 | 46.1 | 45.6 |
| **Ether extract** | 26.1 | 27.0 | 24.5 |
| **Ash** | 6.3 | 5.9 | 6.9 |
| **Gross energy (Mj/kg as fed)** | 22.8 | 23.3 | 22.3 |

**Supplementary Material 1.** Proximate composition of the experimental diets (g/100g as fed, unless otherwise stated). Feed samples were ground using a cutting mill (MLI 204; Bühler AG, Uzwil, Switzerland) and analyzed for DM (AOAC #934.01), CP (AOAC #984.13) and ash (AOAC #942.05) contents according to AOAC International; EE (AOAC #2003.05) was analyzed according to AOAC International. The GE content was determined using an adiabatic calorimetric bomb (C7000; IKA, Staufen, Germany). C: control diet; I: insect meal diet; P: poultry by-products meal diet.

| **INGREDIENTS** | **C** | **I** | **P** |
| --- | --- | --- | --- |
| Fish meal | 27.3 | 11.6 | 11.7 |
| Pig blood meal | 12.1 | 11.7 | 11.7 |
| Poulty by-product meal | 0.0 | 0.0 | 14.8 |
| *Hermetia illucens* meal | 0.0 | 10.1 | 0.0 |
| Fish oil | 11.9 | 11.8 | 11.8 |
| Wheat meal | 7.9 | 10.9 | 7.8 |
| Pea meal | 7.0 | 5.3 | 7.1 |
| Soybean protein concentrate | 6.2 | 11.6 | 6.2 |
| Soybean meal | 6.2 | 6.5 | 6.2 |
| Guar meal | 5.9 | 3.1 | 6.3 |
| Soybean oil | 5.5 | 5.5 | 5.5 |
| Camelina oil | 3.7 | 3.7 | 3.7 |
| Wheat gluten | 3.5 | 3.9 | 3.1 |
| Hydrolyzed fish protein | 1.0 | 1.0 | 1.0 |
| Vit/Min premix | 0.9 | 0.9 | 0.9 |
| Monoammonium Phosphate | 0.0 | 1.3 | 1.2 |
| HCL Lysine | 0.0 | 0.2 | 0.0 |
| DL-methionine | 0.4 | 0.5 | 0.5 |
| Emulsifer (E484) | 0.3 | 0.3 | 0.3 |
| Stay C 35% | 0.1 | 0.1 | 0.1 |
| Antioxidant premix | 0.1 | 0.1 | 0.1 |
| Milk serum protein concentrates | 0.02 | 0.02 | 0.02 |

**Supplementary Material 2.** Ingredients (% as fed) of the experimental diets. Naturalleva (VRM srl, Verona, Italy) provided all ingredients and extruded the feeds. C: control diet; I: insect meal diet; P: poultry by-products meal diet.
