# Supplementary material for: On the Compatibility of Fish Meal Replacements in Aquafeeds for Rainbow Trout. A Combined Metabolomic, Proteomic and Histological Study
Source: Front Physiol. 2022 Jun 29;13:920289. doi: 10.3389/fphys.2022.920289 (PMC9276982; doi:10.3389/fphys.2022.920289)
Supplement: Supplementary file 6 [file Table7.DOCX]

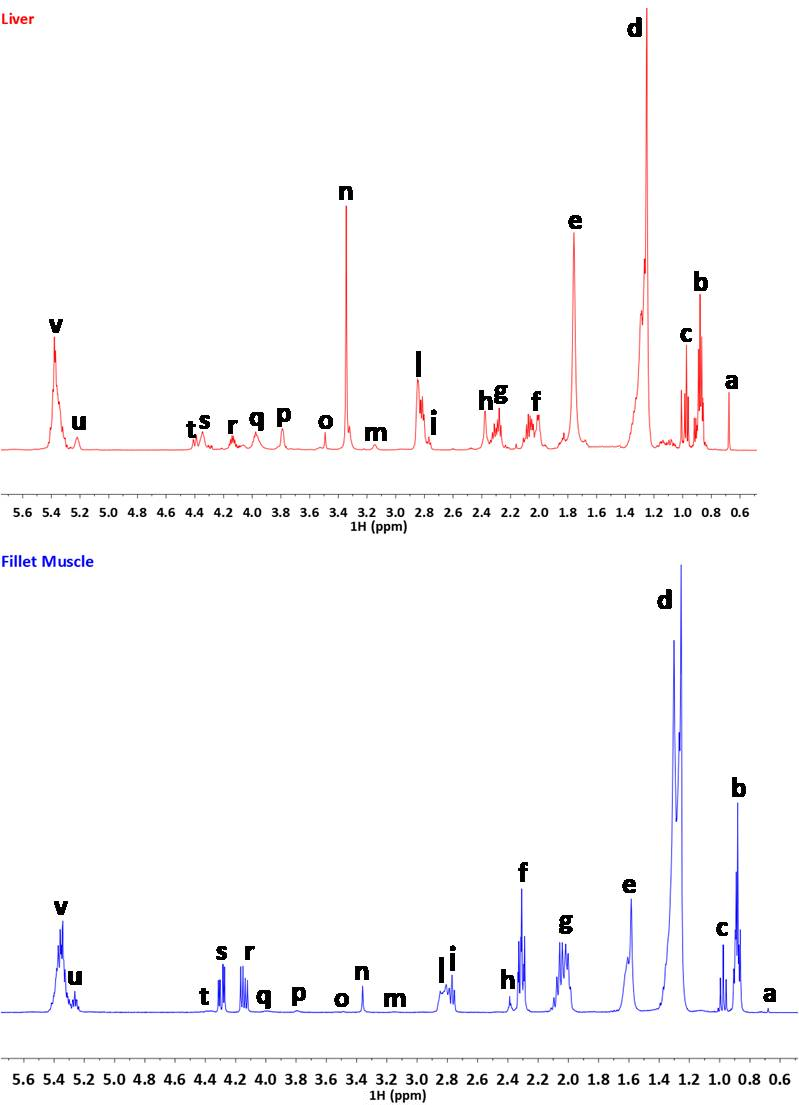


**Supplementary Material 9**. Representative 1D ^1^H NMR lipid profiles extracted by rainbow trout liver (red) and fillet muscle (blue). Letters refer to the assignments reported in the **Supplementary Material 10**.

|  | **Chemical shift range (ppm)** | **Lipid Compound** | **Type of proton** |
| --- | --- | --- | --- |
| A | 0.65-0.69 (s) | **Cholesterol** | -C**H_3_** position 18 |
| B | 0.84-0.93 (t) | **ω6 and ω9 FA** | -C**H_3_** |
| C | 0.95-1.00 (t) | **ω3 FA** | -C**H_3_** |
| D | 1.20-1.40 (m) | **Acyl groups of all FA** | -(C**H_2_**)n |
| E | 1.6-1.8 (broad) | FA Acyl groups and residual water | -OCO-CH_2_-C**H_2_** |
| F | 1.9-2.1 (m) | **All FA diacyl groups** | -C**H_2_**-CH=CH- |
| G | 2.25-2.30 (dt) | **All FA except DHA** | -OCO-C**H_2_**- |
| H | 2.35-2.40 (dt) | **DHA** | -OCO-C**H_2_**- |
| I | 2.73-2.79 (t) | **DUFA** | CH-C**H_2_**-CH |
| L | 2.79-2.84 (m) | **ω3 and ω6 PUFA** | CH-C**H_2_**-CH |
| M | 3.10-3.20 (s) | **2'PE** | O-C**H_2_**-CH_2_-N(PE) |
| N | 3.28-3.40 (s) | **PC** | N(C**H_3_**)_3_ PC |
| O | 3.77-3.81 (broad) | 2'PC | O-C**H_2_**-CH_2_-N(PC) |
| P | 3.90-4.02 (broad) | PC/PE | sn-3 (PC/PE) |
| Q | 4.06-4.09 (broad) | 1'PE | O-CH_2_-C**H_2_**-N(PE) |
| R,S | 4.09-4.32 (m) | **sn-1,3 TAGs** | RO**CH_2_**-CH(OR')-**CH_2_**OR'' |
| T | 5.19-5.24 (broad) | PC/PE | sn-1 (PC/PE) |
| U | 5.24-5.30 (m) | **sn-2 TAGs** | ROCH_2_-C**H**(OR')-CH_2_OR'' |
| V | 5.35-5.45 (m) | **UFA** | -C**H**=C**H**- |

**Supplementary Material 10.** ^1^H NMR assignments of lipids extracted by rainbow trout liver and fillet muscle. Signals selected for subsequent relative quantifications (buckets) are reported in bold. FA = Fatty acids; SFA = Saturated fatty acids; UFA = Unsaturated fatty acids; MUFA = Monounsaturated fatty acids; PUFA = Polyunsaturated fatty acids; TAGs = Triacylglycerols; PC = Phosphatidylcholine; PE = Phosphatidylethanolamine; DHA = docosahexaenoic acid; DUFA = diunsaturated fatty acids.
