# Supplementary material for: On the Compatibility of Fish Meal Replacements in Aquafeeds for Rainbow Trout. A Combined Metabolomic, Proteomic and Histological Study
Source: Front Physiol. 2022 Jun 29;13:920289. doi: 10.3389/fphys.2022.920289 (PMC9276982; doi:10.3389/fphys.2022.920289)
Supplement: Supplementary file 8 [file Table5.DOCX]

**NMR extraction protocol**

Approximately 150 mg of powdered frozen tissue sample was weighted in a 2 ml Eppendorf. As a first step, 600 μL of methanol and 128 μL of water were added, then vortexed for 60 s. In a second step, 600 μL of chloroform and 300 μL of water were added, vortexed for 60 s and left on ice for 10 min. Mixtures were then centrifuged for 10 min at 3000 rpm at 4 °C to enhance partition. Polar (800 μL) and nonpolar (300 μL) layers were separately collected. The nonpolar (chloroformic) fractions were dried under N2 gas flow while the polar extracts were lyophilized (Cinquepascal s.r.l., Milano, Italy, mod. Lio 2000P). The dried lipid extracts were resuspended in 900 μL of deuterated chloroform (CDCl3 99.8% atom D) containing 0.1 % TMS as internal standard (Cambridge Isotope Laboratories, Inc., Tewksbury, MA, USA). Lyophilized polar extracts were resuspended in 900 μL of 50 mM Phosphate Buffer in D2O (99.9% atom D, Sigma Aldrich, St. Louis, MO, USA) with addition of 0.01 mM of TMSP (3-trimethylsilylpropionic acid, 98% D, Cambridge Isotope Laboratories, Inc.). For both polar and lipid extracts, an aliquot of 800 μL was finally transferred in a 5mm NMR tube.
