# Supplementary material for: On the Compatibility of Fish Meal Replacements in Aquafeeds for Rainbow Trout. A Combined Metabolomic, Proteomic and Histological Study
Source: Front Physiol. 2022 Jun 29;13:920289. doi: 10.3389/fphys.2022.920289 (PMC9276982; doi:10.3389/fphys.2022.920289)
Supplement: Supplementary file 11 [file Table6.DOCX]

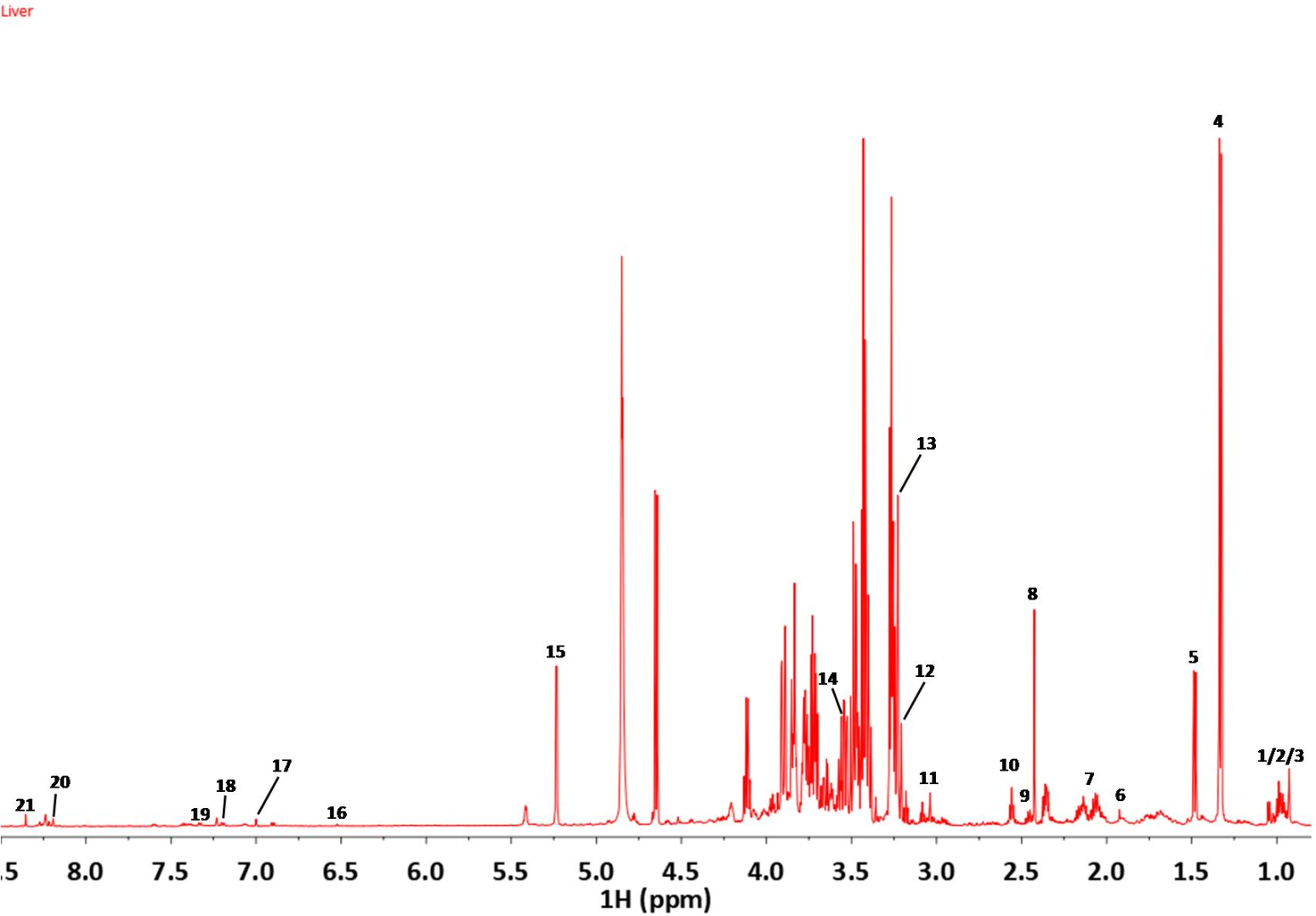


**Supplementary Material 7.** Representative 1D ^1^H NMR polar profile extracted by rainbow trout liver. Letters refer to the assignments reported in the **Supplementary Material 8.**

| **#** | **Compound** | **Assignment** | **δ ^1^H ppm (multiplicity)** | **Compound ID (KEGG)** |
| --- | --- | --- | --- | --- |
| 1 | Leucine | **δ-CH_3_** | **0.96 (d)** | [C00123](http://www.genome.jp/dbget-bin/www_bget?C00123) |
|  |  | δ-CH_3_ | 1.71 (m) |  |
|  |  | α-CH_3_ | 3.75 (t) |  |
| 2 | Isoleucine | **δ-CH_3_** | **0.94 (t)** | [C00407](http://www.genome.jp/dbget-bin/www_bget?C00407) |
|  |  | γ-CH_3_ | 1.01 (d) |  |
|  |  | α-CH | 3.67 (d) |  |
| 3 | Valine | γ-CH_3_ | 0.99 (d) |  |
|  |  | **γ-CH_3_** | **1.05 (d)** | [C00183](http://www.genome.jp/dbget-bin/www_bget?C00183) |
|  |  | α-CH | 3.59 (d) |  |
| 4 | Lactate | **CH_3_** | **1.33 (d)** | [C00186](http://www.genome.jp/dbget-bin/www_bget?C00186) |
|  |  | α-CH | 4.11 (q) |  |
| 5 | Alanine | CH_3_ | **1.48 (d)** | [C00041](http://www.genome.jp/dbget-bin/www_bget?C00041) |
|  |  | CH | 3.78 (q) |  |
| 6 | Acetate | **CH_3_** | **1.92 (s)** | [C00033](http://www.genome.jp/dbget-bin/www_bget?C00033) |
| 7 | Proline | **4- CH_2_** | **1.96-2.11 (m)** | C00148 |
| 8 | Succinate | **CH_2_** | **2.42 (s)** | C00042 |
| 9 | Glutamate | **γ-CH_2_** | **2.46 (m)** | C00025 |
|  |  | α-CH | 3.78 (q) |  |
| 10 | β-alanine | **α-CH_2_** | 2.55 (t) | C00099 |
|  |  | β-CH_2_ | 3.18 (t) |  |
| 11 | Creatine/creatine-phosphate | **CH_3_** | **3.04 (s)** | C00300/C02305 |
| 12 | Choline | **CH_3_** | **3.21 (s)** | C00114 |
| 13 | Betaine | **CH_2_** | **3.27 (s)** | C00719 |
| 14 | Glycine | **CH_2_** | **3.56 (s)** | C00037 |
| 15 | Glucose | **CH_2_** | **5.24 (d)** | C00031 |
| 16 | Fumarate | **CH** | **6.52 (s)** | C00122 |
| 17 | Histidine/methylhistidine | CH_3_ | 3.82 (s) | C00135/C00152 |
|  |  | **4’-CH** | **7.09 (s)** |  |
|  |  | 2’-CH | 8.03 (s) |  |
| 18 | Tyramine/tyrosine | 3’,5’-CH | 6.90 (d) | C00483/C00082 |
|  |  | **2’,6’-CH** | **7.20 (d)** |  |
| 19 | Phenylalanine | **2’,6’-CH** | **7.33 (d)** | C00079 |
|  |  | 4’-CH | 7.38 (t) |  |
|  |  | 3’,5’-CH | 7.43 (t) |  |
|  |  | C_5_H (ring) | 7.60 (dd) |  |
|  |  | C_2_H (ring) | 8.94 (d) |  |
| 20 | Formate | **CH** | **8.36 (s)** | C00058 |
| 21 | Adenosine monophosphate | 1’-CH | 6.18 (d) | C00020/C00130 |
|  |  | 2-CH | 8.24 (s) |  |
|  |  | **8-CH** | **8.42 (s)** |  |

**Supplementary Material 8.** ^1^H NMR assignments of main liver polar metabolites extracted by rainbow trout liver. The signals selected for relative quantifications (buckets) are reported in bold.
